# Exploring the Impact of Subclinical Hypothyroidism on Hyperpigmentation: A Rare Presentation in a 42-Year-Old Woman

**DOI:** 10.7759/cureus.60708

**Published:** 2024-05-20

**Authors:** Bracha Smith, Chiya Abramowitz, Alia Silkov, Martin Kay

**Affiliations:** 1 Dermatology, Kay Dermatology, Burbank, USA; 2 Internal Medicine, New York Institute of Technology College of Osteopathic Medicine, Glen Head, USA

**Keywords:** levothyroxine, autoimmune, skin conditions, endocrine disorders, sub-clinical hypothyroidism, cutaneous hyperpigmentation

## Abstract

Hyperpigmentation of the skin can occur due to internal and external causes. This case highlights an unusual presentation of generalized acute hyperpigmentation associated with subclinical hypothyroidism in a 42-year-old Indian American woman. After unsuccessful trials of various topical agents, the patient exhibited significant improvement in hyperpigmentation after levothyroxine treatment. Improvements included lightening in the bilateral antecubital fossa, axillae, and neck regions. This case underscores the importance of considering thyroid dysfunction as a potential factor that may contribute to atypical pigmentation disorders.

## Introduction

Hyperpigmentation of the skin can occur from a myriad of causes, both internal and external. External causes include postinflammatory hyperpigmentation from contact allergic or irritant eczema, sun exposure, and trauma (such as from burns). Internal causes can be from any medical condition, leading to skin inflammation, as well as from drug reactions, heavy metal ingestion, and some endocrine abnormalities [1]. Due to the complexity and nuances in the diagnosis and management of skin disorders for patients of color, it is common for these patients to seek dermatological assistance for dyspigmentation [2]. Common treatments include (1) topical lightening agents, (2) chemical peels, (3) oral agents, and (4) laser therapy [2].

It is important to ascertain the underlying cause of the hyperpigmentation, if possible, before initiating such treatment.

Here, we present a case of a patient with generalized acute hyperpigmentation that was difficult to treat but resolved after treating her hypothyroidism.

## Case presentation

**Figure 1:**
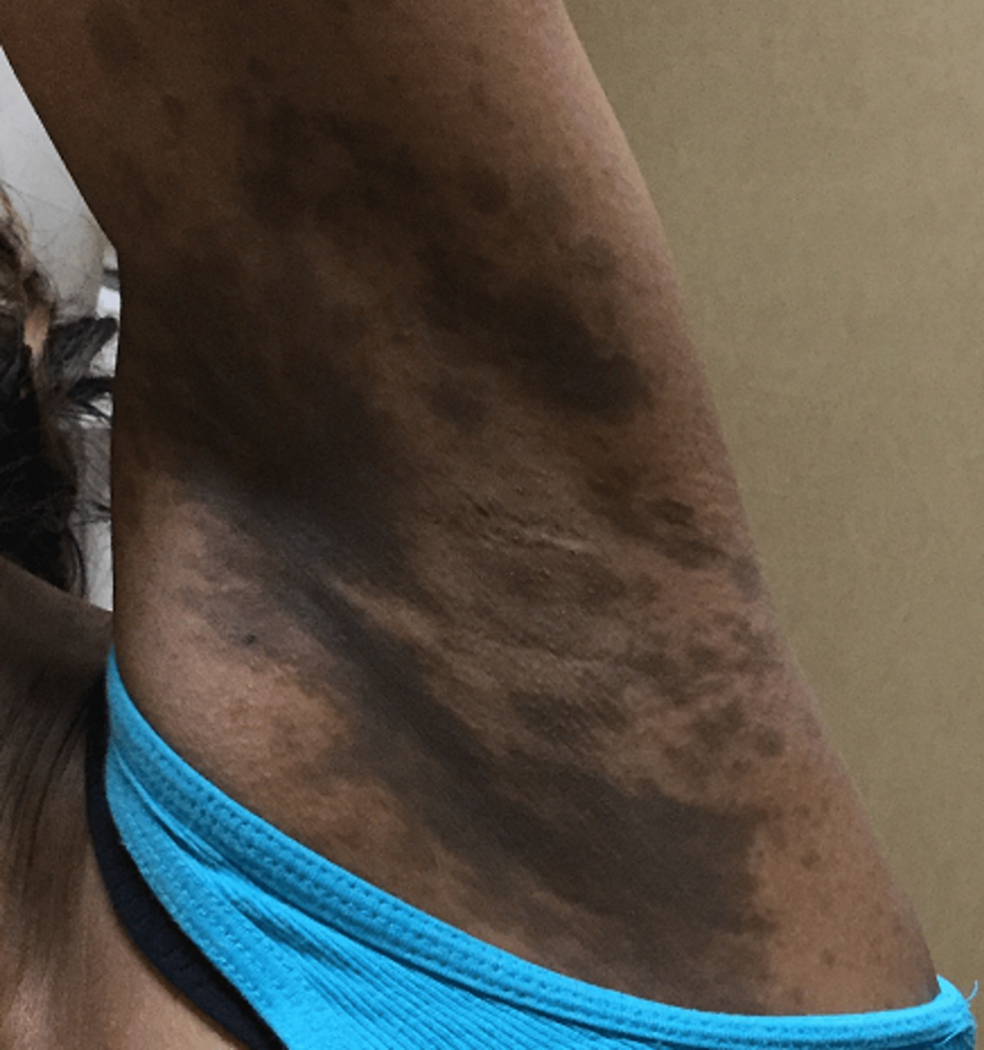
Pre-treatment hyperpigmentation of the left axilla

**Figure 2:**
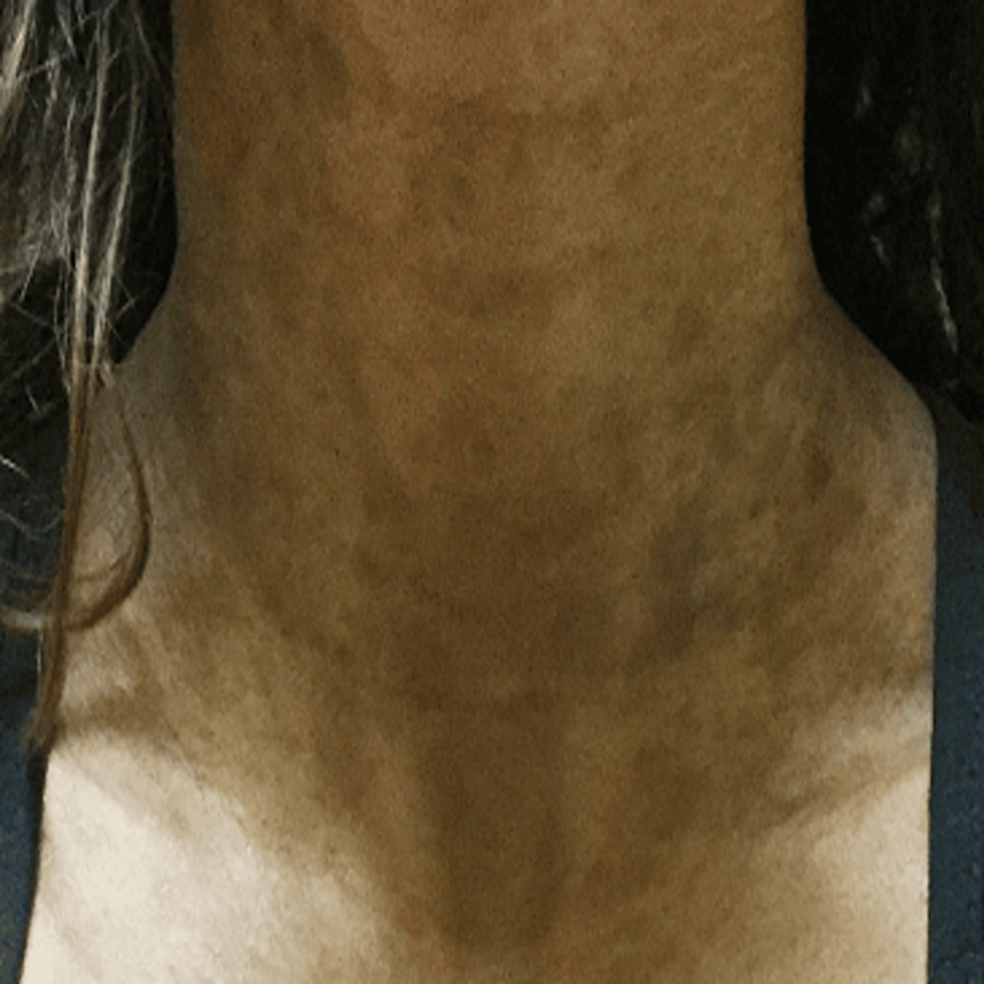
Pre-treatment hyperpigmentation of the neck regions

A 42-year-old Indian American woman with Fitzpatrick skin type IV presented with hyperpigmented black and gray macules scattered on her neck, axillae, arms, and upper chest (Figures 1-2).

**Figure 3:**
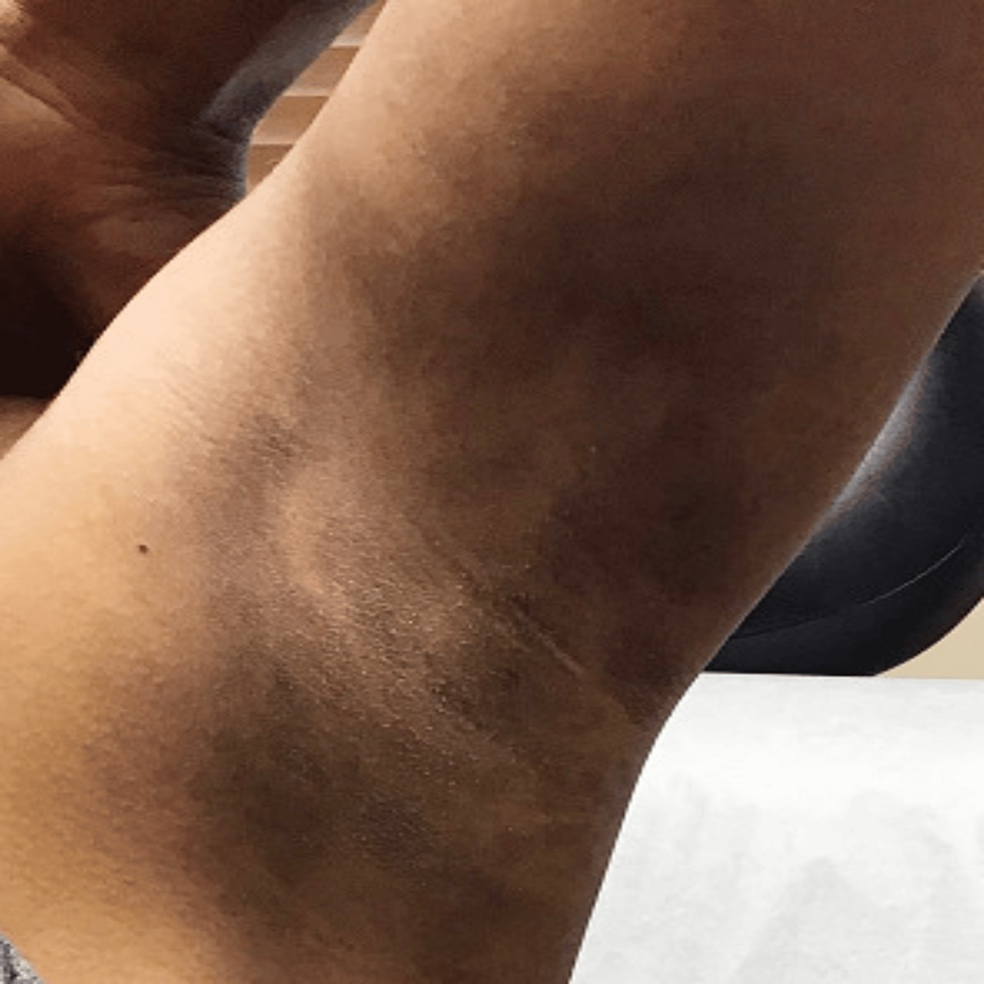
Post-treatment hyperpigmentation of the left axilla

**Figure 4:**
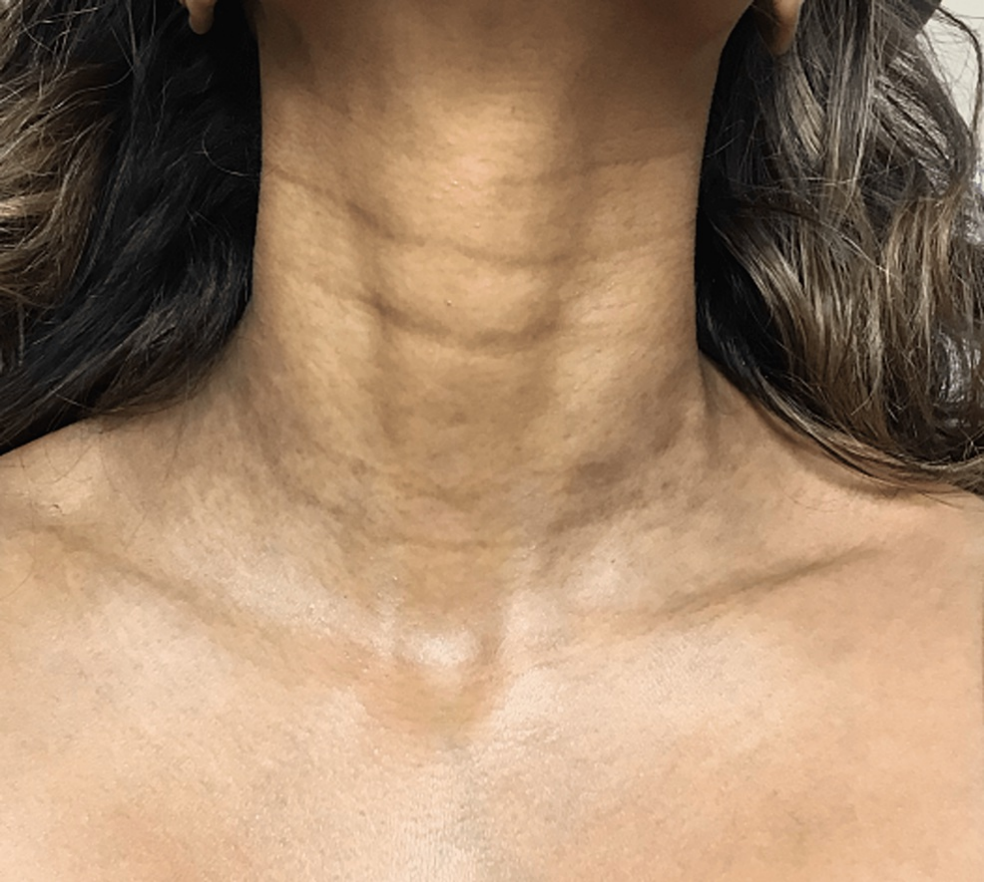
Post-treatment hyperpigmentation of the neck regions

No macules were present on her face, feet, and hands. The patient has a history of asthma, dry skin, eczema, itchy scalp, and allergies. Previously, she had a biopsy of the patches at another practice that revealed generalized inflammatory changes. The physician at the practice prescribed topical hydroquinone 4%, a common depigmenting agent, qd for three months, as the biopsy did not reveal any specific dermatopathological features indicative of a dichromic disease, but the treatment did not alleviate her symptoms. Treatment with topical methimazole cream was initiated and did not lighten the spots significantly as the hyperpigmented macules continued to spread. The patient came to our practice seeking a second opinion. Upon further evaluation and desired improvement, bloodwork revealed subclinical hypothyroidism with a moderately elevated thyroid-stimulating hormone (TSH) level and mildly decreased free T4 level at 7.02 and 1.06, respectively. The patient also had normal thyroid peroxidase antibody (anti-TPO) levels, as well as normal cortisol, and glucose levels. Although the thyroid function tests (TFTs) were borderline abnormal, with no apparent clinical symptoms, her primary care physician began treating her thyroid with levothyroxine at our advice. On follow-up 74 weeks later, both her TFTs and hyperpigmentation significantly improved, with a TSH of 0.78 and free T4 of 1.42 and an 80% reduction in hyperpigmentation with minimal pigmentation present on physical examination. After treatment, her skin in the bilateral antecubital fossa regions had lightened to the point where the pigmentation was hardly noticeable, while the pigmentation in her axillas improved but is still noticeable (Figure 3). Further, the pigmentation in the neck region significantly improved (Figure 4).

## Discussion

The patient presented with hyperpigmentation that is likely attributed to decreased thyroid hormone levels. Thyroid dysfunction can affect many organ systems, including the integumentary system. Aside from systemic autoimmune diseases such as Hashimoto’s thyroiditis or Addison’s disease, pituitary disorders such as secondary hypothyroidism, cases of medication-induced hyperpigmentation, or hemochromatosis, hyperpigmentation in isolated thyroid disorders is reported mainly in hyperthyroidism [3]. There are few cases reported of isolated hypothyroidism causing hyperpigmentation [4,5].

Initially, it was suspected that hyperpigmentation was further exacerbated by the drug used for the intended treatment by the primary practice. However, after ceasing her medication, this cause was ruled out. Other clinically relevant causes of hyperpigmentation related to this case were also ruled out. Additionally, dry skin, atopic dermatitis, and an itchy scalp can all be associated with thyroid conditions [6]. Given the patient’s history of atopic dermatitis, this could have been a contributing factor. However, the cause can be traced back to her mild thyroid abnormality, as the most considerable improvement in pigmentation was noticed after levothyroxine treatment by her primary care physician.

There is a paucity of literature exploring the relationship between subclinical hypothyroidism and hyperpigmentation. Other endocrine diseases associated with pigmentation presentations include the following: Cushing syndrome, Addison’s disease, hypopituitarism, and diabetes mellitus [7]. While certain thyroid disorders such as Hashimoto’s thyroiditis can sometimes co-occur with other autoimmune conditions such as Addison’s disease, which is often associated with hyperpigmentation due to increasing adrenocorticotropic hormone (ACTH) levels [1], this patient had normal thyroid peroxidase antibody (anti-TPO) levels, which would otherwise confirm the diagnosis of Hahimoto’s disease.

Addison's disease often presents with diffuse pigmentation with a preferential occurrence on the mucous membranes and over-pressure points, such as the oral cavity, conjunctiva, and genitalia [1]. Pigmentation caused by Addison’s disease presents with melanin in the subepithelial and basal layers [1]. The patient’s biopsy revealed a mildly atrophic stratum spinosum and patchy hyperpigmentation at the basal layer. Melanophages and macrophages were spread throughout the papillary dermis. Additionally, this patient was normotensive with normal glucose levels, and metabolic syndrome as well as acanthosis nigricans were ruled out, suggesting another etiology for her hyperpigmentation.

The hyperpigmentation was likely caused by her subclinically low thyroid level. Initially, topical lightening creams were given to try to treat the pigmentation. However, it did not lighten the pigmentation as much as desired. However, once the thyroid levels began being treated, in conjunction with topical creams, significant improvement was seen. 

This case is novel due to the relatively low degree of pre-existing hypothyroidism and the large role that it may have played in the development of hyperpigmentation. With a TSH of 7.02 and no clinical symptoms of hypothyroidism, it is difficult for clinicians to attribute skin findings to an asymptomatic condition. This is especially true in a presentation such as this one whereby the patient recently started a new drug treatment. For this reason, determining the etiology of hyperpigmentation is paramount in establishing a timely diagnosis and treatment. As a rare cause of hyperpigmentation, the complex pathology and systemic manifestations of hypothyroidism require clinicians to be mindful and understand its masquerading effects in such presentations. Therefore, it is of utmost importance that clinicians should be aware of the association between hypothyroidism and abnormalities in pigmentation, and it may indicate a larger implication for ordering routine blood work in patients who present with similar patterns of hyperpigmentation.

However, the case does not come without its limitations. Such existing limitations that may have affected the results of this study included the confounding treatments administered to this patient. In particular, when methimazole was initially administered for hyperpigmentation, it may have precipitated hypothyroidism as the drug is commonly used for hyperthyroidism [8].

Overall, this case underscores the importance of considering subclinical hypothyroidism as a potential cause of hyperpigmentation, emphasizing the need to be vigilant in recognizing thyroid dysfunction in patients with unusual pigmentation patterns while also acknowledging potential confounding factors in treatment.

## Conclusions

Although subclinical hypothyroidism is commonly asymptomatic, it may be unexpectedly linked to certain dermatologic findings, such as hyperpigmentation. As seen in this case, such patients may be successfully treated for their underlying thyroid condition in order to treat the secondary skin conditions. Although not typically seen in practice, clinicians should remain mindful and vigilant when managing patients with both hypothyroidism and hyperpigmentation. Further research in similar patient cases may offer valuable insights into understanding pigmentation disorders and their relationship to thyroid dysfunction.
